# Mechanisms underlying differential response to estrogen-induced apoptosis in long-term estrogen-deprived breast cancer cells

**DOI:** 10.3892/ijo.2014.2329

**Published:** 2014-03-06

**Authors:** ELIZABETH E. SWEENEY, PING FAN, V. CRAIG JORDAN

**Affiliations:** Lombardi Comprehensive Cancer Center, Georgetown University Medical Center, Washington, DC, USA

**Keywords:** oxidative stress, estrogen deprivation, breast cancer, insulin-like growth factor receptor, glutathione

## Abstract

Models of long-term estrogen-deprived breast cancer cells are utilized in the laboratory to mimic clinical aromatase inhibitor-resistant breast cancer and serve as a tool to discover new therapeutic strategies. The MCF-7:5C and MCF-7:2A subclones were generated through long-term estrogen deprivation of estrogen receptor (ER)-positive MCF-7 cells, and represent anti-hormone-resistant breast cancer. MCF-7:5C cells paradoxically undergo estrogen-induced apoptosis within seven days of estrogen (estradiol, E_2_) treatment; MCF-7:2A cells also experience E_2_-induced apoptosis but evade dramatic cell death until approximately 14 days of treatment. To discover and define the mechanisms by which MCF-7:2A cells survive two weeks of E_2_ treatment, systematic experiments were performed in this study. The data suggest that MCF-7:2A cells employ stronger antioxidant defense mechanisms than do MCF-7:5C cells, and that oxidative stress is ultimately required for MCF-7:2A cells to die in response to E_2_ treatment. Tumor necrosis factor (TNF) family member activation is also essential for E_2_-induced apoptosis to occur in MCF-7:2A cells; upregulation of TNFα occurs simultaneously with oxidative stress activation. Although the unfolded protein response (UPR) signaling pattern is similar to that in MCF-7:5C cells, it is not sufficient to cause cell death in MCF-7:2A cells. Additionally, increased insulin-like growth factor receptor β (IGF-1Rβ) confers a mechanism of growth and anti-apoptotic advantage in MCF-7:2A cells.

## Introduction

Aromatase inhibitor-resistant breast cancer cells are modeled *in vitro* by long-term E_2_-deprived breast cancer cell lines. The MCF-7:WS8 cell line represents a clone of the estrogen receptor (ER)-positive cell line MCF-7 that is highly sensitive to E_2_-stimulated growth (1). The MCF-7:5C and MCF-7:2A subclones are derived from the parental MCF-7 cell line through long-term E_2_ deprivation (1–4). MCF-7:5C cells express wild-type ER at a higher level than the parental line, and are progesterone receptor (PR)-negative (3). These cells grow in the absence of E_2_, and do not respond to 4-hydroxytamoxifen (4-OHT) (2,3). MCF-7:2A cells can induce expression of PR and express both wild-type (66 kDa) and mutant (77 kDa) ER (4,5). The mutant ER contains a repeat of exons 6 and 7 and cannot bind E_2_ nor anti-estrogens; it is expressed 4- to 10-fold lower than the wild-type ER (6). The total ER level of MCF-7:2A cells is higher than in parental MCF-7 cells, and they also grow in E_2_-free media. 4-OHT and pure anti-E_2_ are able to block their growth (4,5).

In addition to the different responses to anti-E_2_ observed in MCF-7:5C versus MCF-7:2A cells, they also have different apoptotic responses to E_2_. The MCF-7:5C cells undergo apoptosis and die during the first week of E_2_ treatment, whereas the MCF-7:2A cells die later, after two weeks of E_2_ treatment (7). MCF-7:5C cell response to estrogens and anti-estrogens has been extensively studied in our lab; the data show that these cells undergo E_2_-induced apoptosis through mechanisms associated with endoplasmic reticulum stress (ERS) and oxidative stress (8,9). Thus far, there has been less focus on the classification and mechanisms of the MCF-7:2A response.

**Figure 1.**
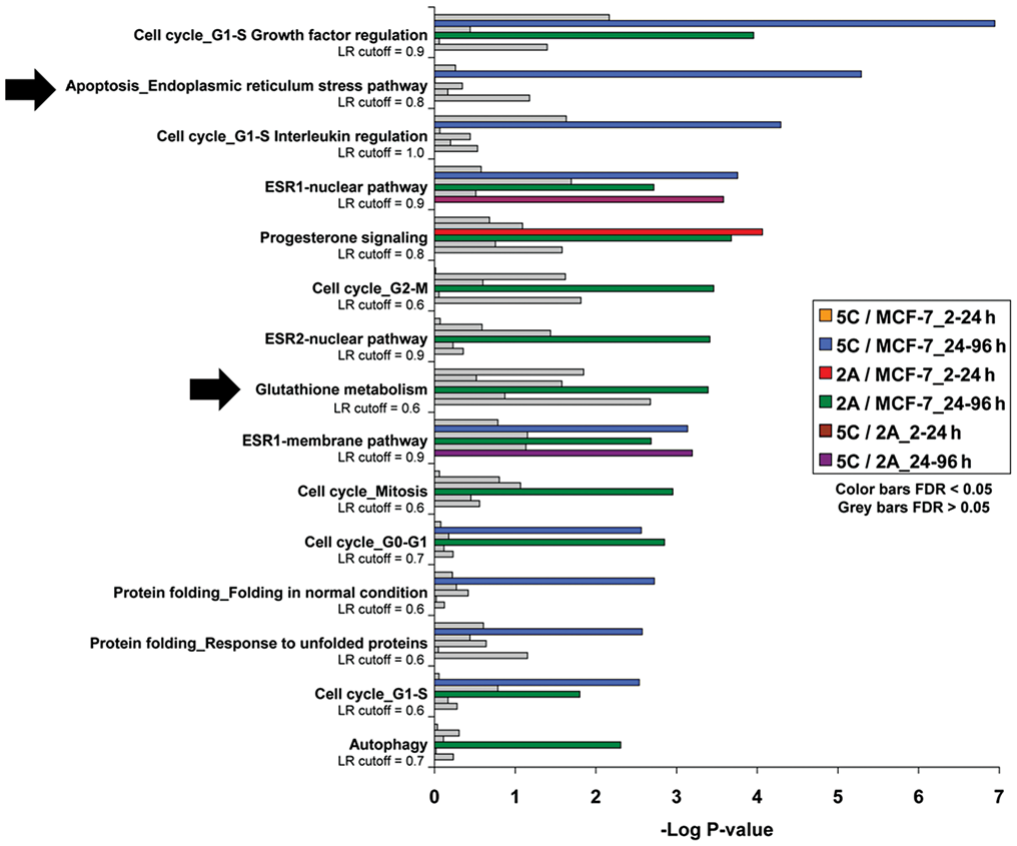
Network enrichment analysis for MCF-7:WS8, MCF-7:5C and MCF-7:2A cells. Global gene arrays were performed to compare activated gene networks associated with 1 nM E_2_ treatment in the cell lines. Genes were analyzed after 2–24 and 24–96 h treatment.

Network enrichment analyses done using gene arrays in timecourse experiments show overexpression of apoptotic- and stress-related pathways in the MCF-7:5C cells after 24–96 h of E_2_ treatment; however, these analyses show the MCF-7:2A cells expressing more genes associated with glutathione metabolism during this time period of E_2_ exposure (Fig. 1). This suggests that the two cell lines respond to E_2_ treatment using different signaling pathways. The MCF-7:5C cells respond by quickly inducing apoptosis, while the anti-oxidant pathway may be more relevant to the MCF-7:2A cells. Experiments were designed to interrogate the apoptotic, stress and antioxidant pathways in both cell lines to distinguish signaling mechanisms in response to E_2_.

The concept of E_2_-induced death is important because of its clinical relevance. A clinical study published in 2009 (10) compared two doses of E_2_ for second-line treatment after breast cancer patients had failed aromatase inhibitor therapy. The authors showed that after long-term anti-hormone therapy, no response is lost with the lower dose of E_2_; overall about 30% of women responded to E_2_ treatment. The goal of this study is to uncover the mechanisms preventing the other 70% of patients from responding, and perhaps find ways to circumvent their resistance. To this end, MCF-7:2A cells were used as a model for E_2_-deprived breast tumors with the ability to evade E_2_-induced apoptosis in the clinic.

## Materials and methods

### Cell culture

All cell lines were cultured in phenol red-free RPMI-1640 media supplemented with 10% charcoal-stripped fetal bovine serum (SFS). Media and treatments were replaced every three days. Estradiol (E_2_) (Sigma-Aldrich, St. Louis, MO, USA), buthionine sulfoximine (BSO) (Sigma-Aldrich), and combinations were dissolved in ethanol and then in media. AG1024 (Calbiochem, San Diego, CA, USA) was dissolved in DMSO and then in media.

### DNA assays

MCF-7:WS8, MCF-7:5C and MCF-7:2A cells were harvested after 7 or 14 days treatment with vehicle (0.1% ethanol), E_2_ (10^−9^ mol/l, 1 nM), BSO (10^−4^ mol/l, 100 *μ*M), or E_2_ (1 nM) + BSO (100 *μ*M). DNA content was measured as previously described (11).

### Western blot analysis

Total MAPK (#9102), phosphorylated MAPK (#9101), total AKT (#9272), phosphorylated AKT (#4051L), total eIF2α (#9722S), phosphorylated eIF2α (#9721S), and IRE1α (#3294S) antibodies were all purchased from Cell Signaling Technology (Beverly, MA, USA). IGF-1Rβ antibody (sc-713) was purchased from Santa Cruz Biotechnology (Santa Cruz, CA, USA). β-actin loading control antibody (A5441) was purchased from Sigma-Aldrich. Proteins were harvested from cells using cell lysis buffer (Cell Signaling Technology) supplemented with Protease Inhibitor Cocktail Set I and Phosphatase Inhibitor Cocktail Set II (Calbiochem). Bicinchoninic acid (BCA) assay was used to quantify total protein content (Rio-Rad Laboratories, Hercules, CA, USA). Protein (50 *μ*g) was probed and visualized as previously described (11).

### Cell cycle analysis

MCF-7:2A cells were cultured in dishes and treated with vehicle (0.1% ethanol) or E_2_ (10^−9^ mol/l, 1 nM). Cells were harvested after 24 h, fixed in 75% ethanol on ice, stained with propidium iodide and sorted using FACS flow cytometry (Becton Dickinson, San Jose, CA, USA). Results were analyzed using CellQuest software.

### RT-PCR

Cells were harvested using TRIzol, and RNA was isolated using RNeasy mini kit (Qiagen, Valencia, CA, USA). RNA was reverse transcribed to cDNA using a kit (Applied Biosystems, Foster City, CA). SYBR-Green (Applied Biosystems) was used for quantitative real-time polymerase chain reaction (RT-PCR) in a 7900HT Fast Real-Time PCR system (Applied Biosystems).

### Glutathione assay

Cells were harvested and de-proteinized with 5% 5-sulfosalicylic acid solution (SSA) (Sigma-Aldrich). Total glutathione [reduced glutathione (GSH) plus glutathione disulfide (GSSG)] was measured spectroscopically at 412 nm using a Glutathione Assay Kit (CS0260, Sigma-Aldrich) and the manufacturer’s instructions.

### ROS assay

MCF-7:2A cells were harvested, stained with 10^−6^ mol/l (1 *μ*M) CM-H2DCFDA (Invitrogen, Eugene, OR, USA), and analyzed for ROS fluorescence using flow cytometry.

### Statistical analysis

Values reported are means ± standard deviation (SD). Significant differences were found by Student’s t-test. P-values <0.05 were considered to indicate a statistically significant difference.

## Results

### MCF-7:2A initial response to E_2_

**Figure 2.**
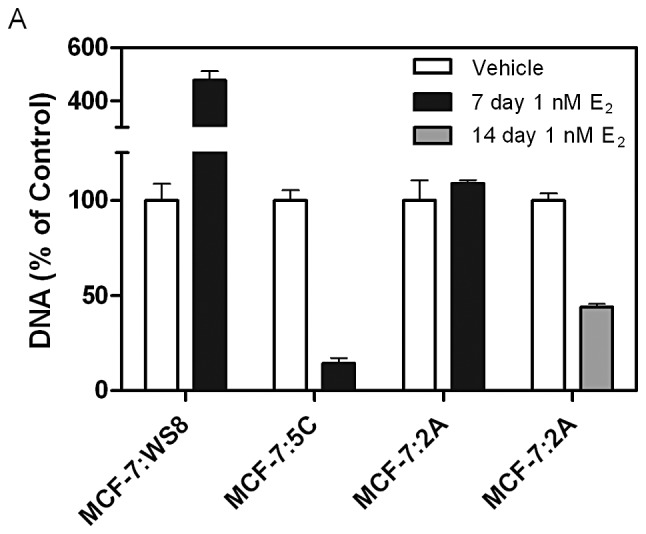

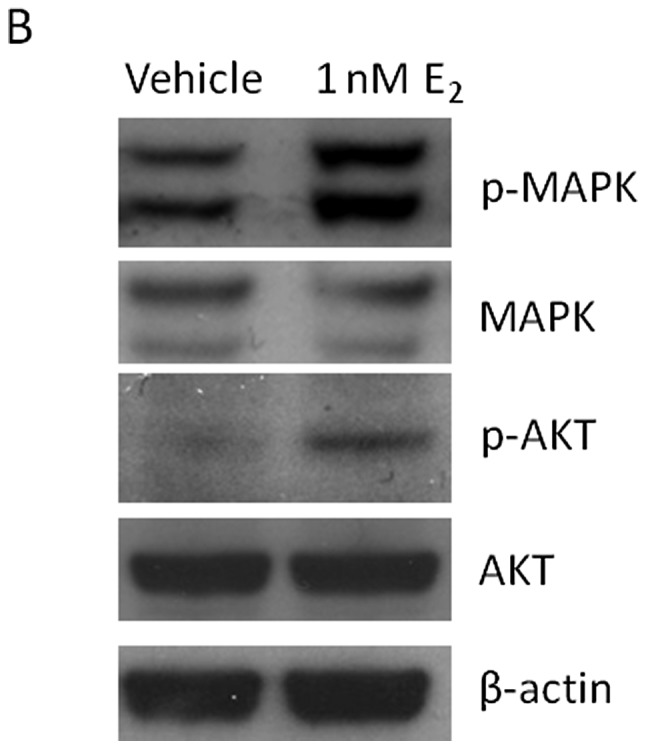

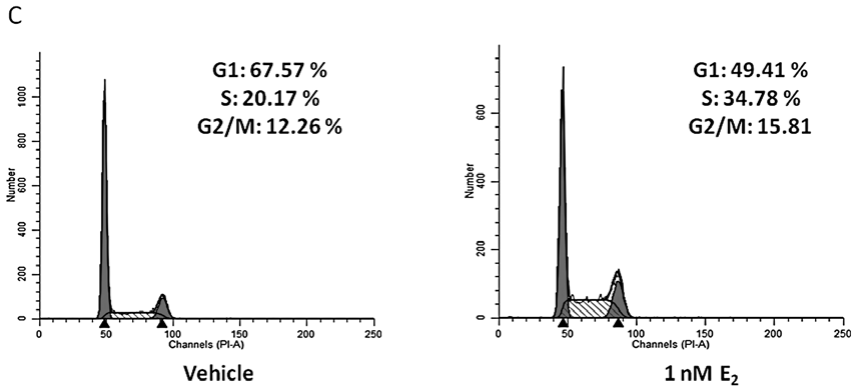
MCF-7:2A growth response to E_2_. (A) DNA was measured from MCF-7:WS8, MCF-7:5C and MCF-7:2A cells after 7 or 14 days treatment with vehicle or 1 nM E_2_. Values are normalized to vehicle-treated cells. Means represent samples in triplicate. (B) MAPK and AKT growth pathway protein levels were measured by western blot analysis after 24 h vehicle or 1 nM E_2_ treatment. β-actin was used as a loading control. (C) Cell cycle analysis was performed after 24 h vehicle or 1 nM E_2_ treatment.

The MCF-7:WS8, MCF-7:5C and MCF-7:2A cell lines respond differently to 10^−9^ mol/l (1 nM) E_2_. In the presence of 1 nM E_2_, MCF-7:WS8 cells are stimulated to proliferate over 7 days, whereas MCF-7:5C cells are killed by this time point (Fig. 2A). MCF-7:2A cell growth is unaffected by the presence of E_2_ after one week, but their DNA is reduced by 50% after the second week of treatment (Fig. 2A). Interestingly, MCF-7:2A cells are initially stimulated to proliferate in response to E_2_. After 24 h-treatment with 1 nM E_2_, both the mitogen-activated protein kinase (MAPK) and serine/threonine protein kinase Akt (AKT) pathways are activated, as shown by an increase in phosphorylated MAPK (p-MAPK) and phosphorylated AKT (p-AKT) proteins, respectively (Fig. 2B). Further, MCF-7:2A cells treated with E_2_ for 24 h show an increase in the percentage of dividing cells compared with vehicle treatment (34.78 versus 20.17%), illustrated by S-phase in cell cycle analysis (Fig. 2C).

### MCF-7:5C and MCF-7:2A UPR

**Figure 3.**
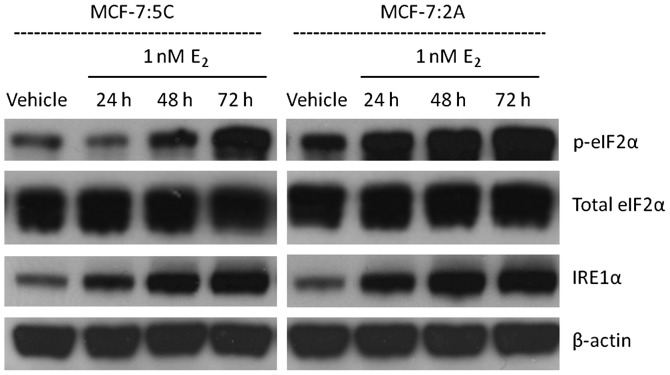
MCF-7:5C and MCF-7:2A UPR. Cell lines were probed for UPR-related proteins after treatment with vehicle or 1 nM E_2_ for 24, 48 and 72 h. β-actin was used as a loading control.

To determine whether the different biological effects observed in MCF-7:5C and MCF-7:2A cells is due to different patterns of the unfolded protein response (UPR), proteins associated with the UPR were measured over a 72 h timecourse. Two markers of the UPR, phosphorylated eIF2α (p-eIF2α) and IRE1α, were visualized by western blot analysis in MCF-7:5C and MCF-7:2A cells in the presence of vehicle and 1 nM E_2_ (Fig. 3). p-eIF2α is directly downstream of protein kinase RNA-like endoplasmic reticulum kinase (PERK), a sensor which initiates UPR. Both cell lines show an increase in the protein expression of p-eIF2α and IRE1α by 72 h of E_2_ treatment, indicating activated UPR. Though MCF-7:2A cells show a slightly higher basal p-eIF2α level, no differences in UPR activation can be seen between the two cell lines.

### MCF-7:5C and MCF-7:2A estrogen-induced apoptosis

**Figure 4.**
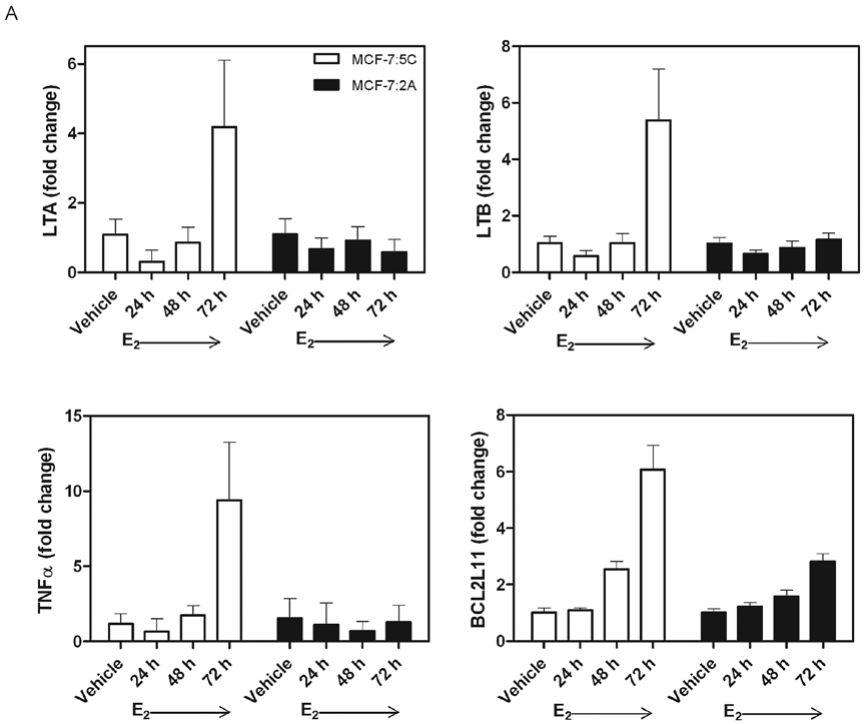

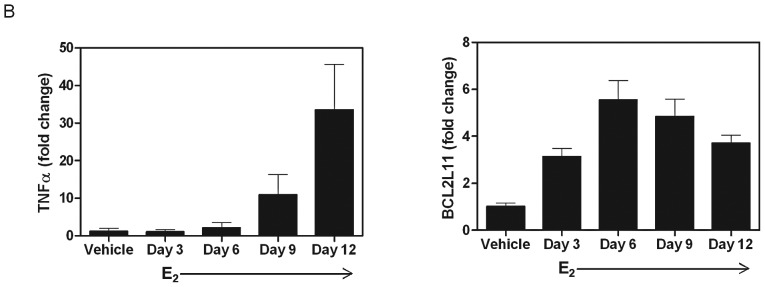
Apoptosis-related genes in MCF-7:5C (white bars) and MCF-7:2A (black bars) cells. (A) MCF-7:5C and MCF-7:2A cells were treated with vehicle or 1 nM E_2_ for 24, 48 and 72 h. LTA, LTB, TNFα and BCL2L11 mRNA levels were measured using RT-PCR. 36B4 was used as an internal control. (B) MCF-7:2A cells were treated with vehicle or 1 nM E_2_ for 3, 6, 9 and 12 days. TNFα and BCL2L11 mRNA levels were then measured using RT-PCR. 36B4 was used as an internal control. Means represent at 7 to 18 replicates.

To determine whether MCF-7:2A cells experience apoptosis through the same mechanism as MCF-7:5C cells, RT-PCR was used to quantify mRNA levels of apoptosis-related genes. MCF-7:5C cells noticeably upregulate LTA (4.19±1.92 fold change), LTB (5.39±1.82), TNFα (9.40±3.86), and BCL2L11 (6.06±0.87) after 72 h of E_2_ treatment, while MCF-7:2A cells show no major changes during this time period (Fig. 4A). MCF-7:2A cells were then treated with E_2_ for a longer time period to measure apoptosis-related genes during the time when they appear to die. MCF-7:2A cells increase both TNFα (33.55±12.09 fold change) and BCL2L11 (3.71±0.35 fold change) after 12 days of 1 nM E_2_ treatment (Fig. 4B). The upregulated apoptosis-related genes correspond to the time when cell death is most apparent in both cell lines, during week one in MCF-7:5C cells, and during week two in MCF-7:2A cells.

### MCF-7:5C and MCF-7:2A oxidative stress

**Figure 5.**
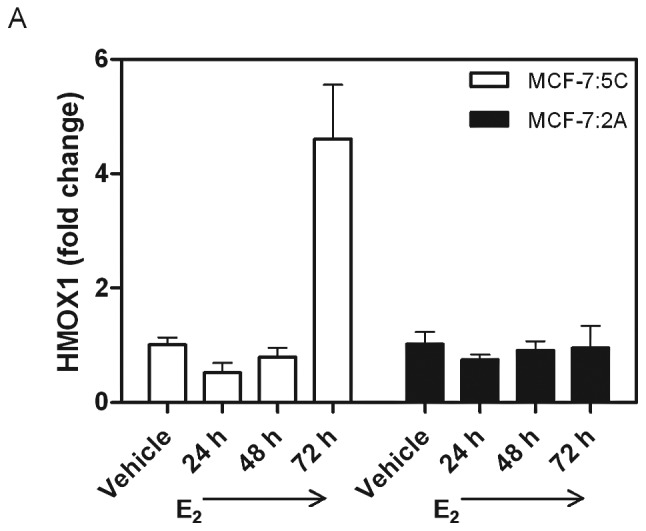

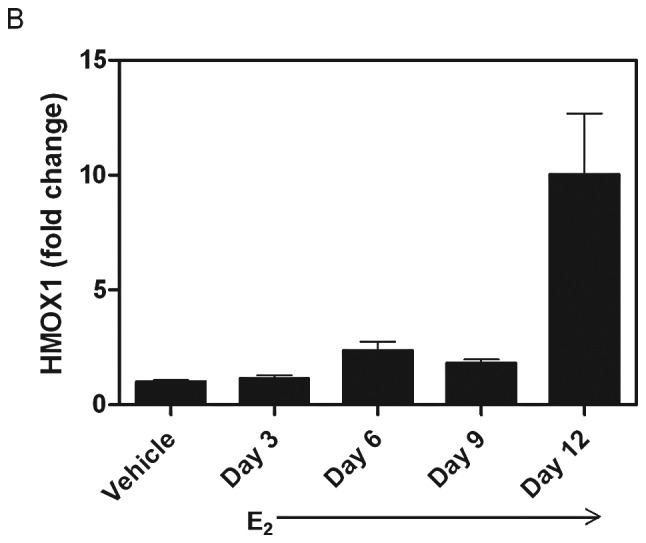
MCF-7:5C and MCF-7:2A HMOX1 regulation. (A) MCF-7:5C and MCF-7:2A cells were treated with vehicle or 1 nM E_2_ for 24, 48 and 72 h; HMOX1 mRNA was measured using RT-PCR. 36B4 was used as an internal control. Mean represents 18 replicates. (B) MCF-7:2A cells were treated with vehicle or 1 nM E_2_ for 3, 6, 9 and 12 days; HMOX1 mRNA was measured using RT-PCR. 36B4 was used as an internal control. Means represent at least 8 replicates.

Heme oxygenase 1 (HMOX1) was used as an indicator to illustrate when MCF-7:5C and MCF-7:2A cells experience oxidative stress. After 72 h of 1 nM E_2_ treatment, HMOX1 mRNA was increased 4.61-fold in MCF-7:5C cells (Fig. 5A), suggesting this cell line undergoes oxidative stress at this time point. MCF-7:2A cells did not generate an upregulation of HMOX1 mRNA until 12 days of 1 nM E_2_ treatment when it increased 10.03-fold (Fig. 5B), suggesting an earlier protective mechanism inherent in these cells to prevent oxidative stress longer than MCF-7:5C cells.

**Figure 6.**
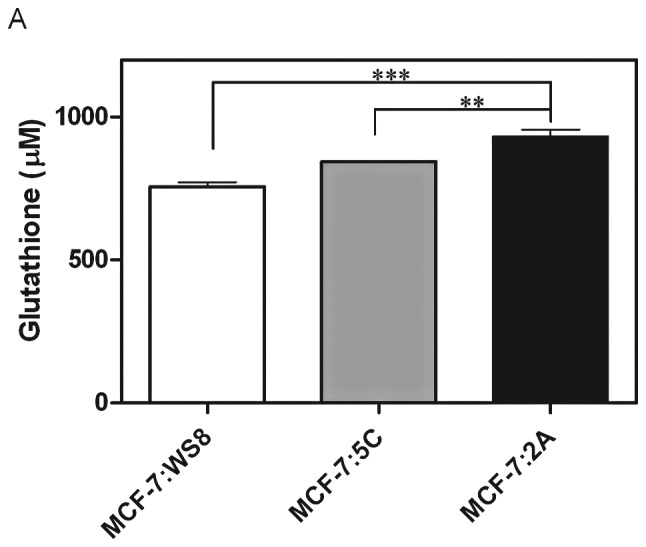

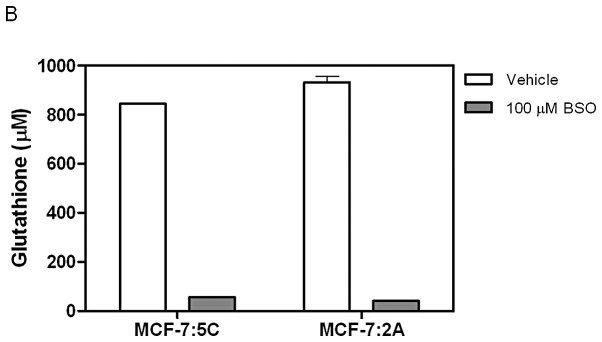

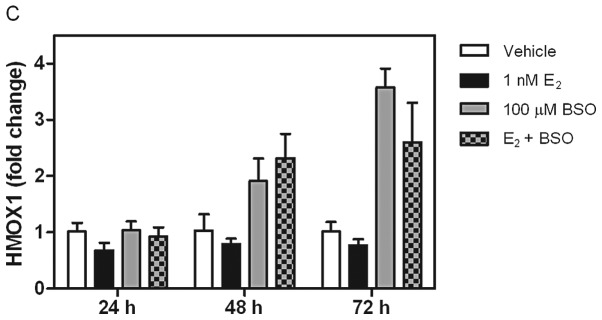

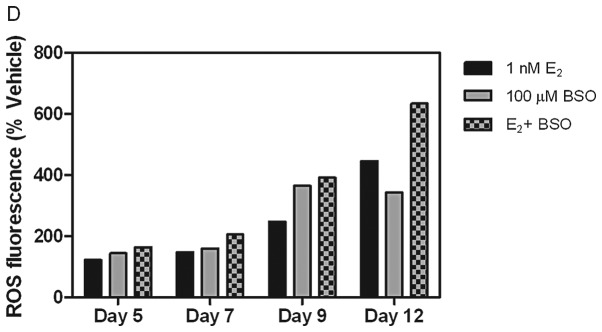

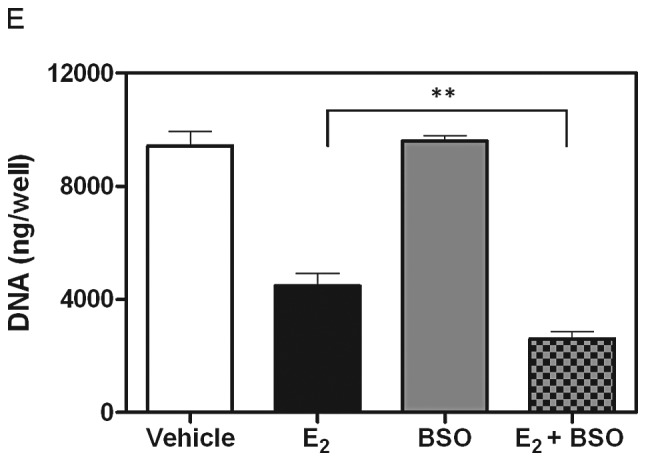
MCF-7:2A oxidative stress and glutathione. (A) Total basal glutathione (GSSG+GSH) levels were measured in MCF-7:WS8, MCF-7:5C and MCF-7:2A cells. Means represent samples in triplicate. (B) Total glutathione in MCF-7:5C and MCF-7:2A cells were quantified after 72 h treatment of vehicle or 100 *μ*M BSO. Means represent samples in triplicate. (C) MCF-7:2A cells were treated for 24, 48 and 72 h with either vehicle, 1 nM E_2_, 100 *μ*M BSO or 1 nM E_2_ + 100 *μ*M BSO; HMOX1 mRNA was measured using RT-PCR. 36B4 was used as an internal control. Means represent at least 8 replicates. (D) MCF-7:2A were subjected to the aforementioned treatments for 5, 7, 9 and 12 days, and ROS levels were measured. Data are normalized to vehicle treatment. (E) MCF-7:2A cells were treated likewise, and DNA was harvested and quantified after two weeks. Means represent samples in triplicate. ^**^P<0.01, ^***^P<0.001.

Glutathione is a potent antioxidant and was quantified in MCF-7:5C and MCF-7:2A cells to illustrate a potential protective mechanism in MCF-7:2A cells against oxidative stress (Fig. 6A). In fact, MCF-7:2A cells have significantly more basal glutathione than do MCF-7:WS8 and MCF-7:5C cells (Fig. 6A). Buthionine sulfoximine (BSO) is a synthetic amino acid that blocks glutathione synthesis by inhibiting γ-glutamylcysteine synthetase. BSO (100 *μ*M) dramatically decreases glutathione levels in both MCF-7:5C and MCF-7:2A cells (Fig. 6B). To ask the question of whether glutathione is protecting MCF-7:2A cells from oxidative stress and E_2_-induced apoptosis, HMOX1 was measured following treatment with vehicle, 1 nM E_2_ alone, 100 *μ*M BSO alone, and 1 nM E_2_ + 100 *μ*M BSO after 24, 48 and 72 h (Fig. 6C). MCF-7:2A cells show increased HMOX1 mRNA at 72 h after treatment with 100 *μ*M BSO and 1 nM E_2_ + 100 *μ*M BSO (3.57±0.36 and 2.60±0.70 fold change, respectively), suggesting a protective role of glutathione in these cells. Reactive oxygen species (ROS) increased 634% over vehicle in MCF-7:2A cells after 12 days of the combination treatment (Fig. 6D). Furthermore, 1 nM E_2_ + 100 *μ*M BSO treatment caused a significant decrease in DNA after 14 days treatment (Fig. 6E), suggesting that oxidative stress is a key factor in determining E_2_-induced MCF-7:2A cell death.

### MCF-7:5C and MCF-7:2A IGFR

**Figure 7.**
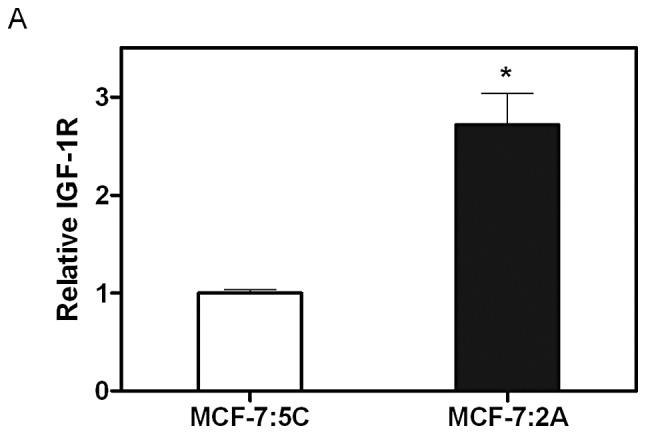

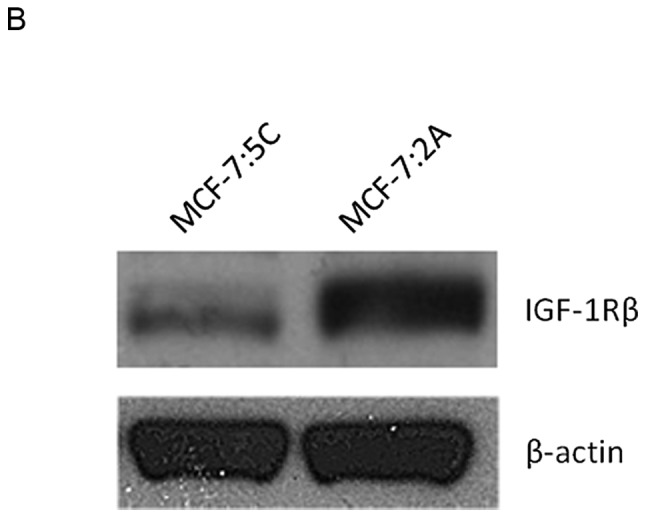

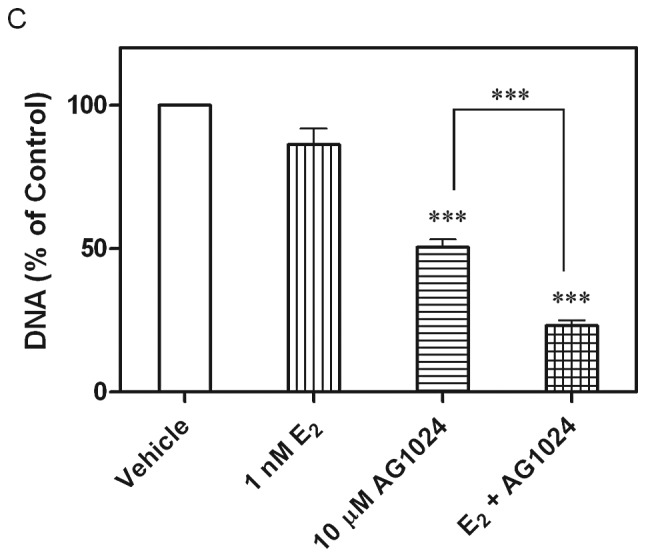

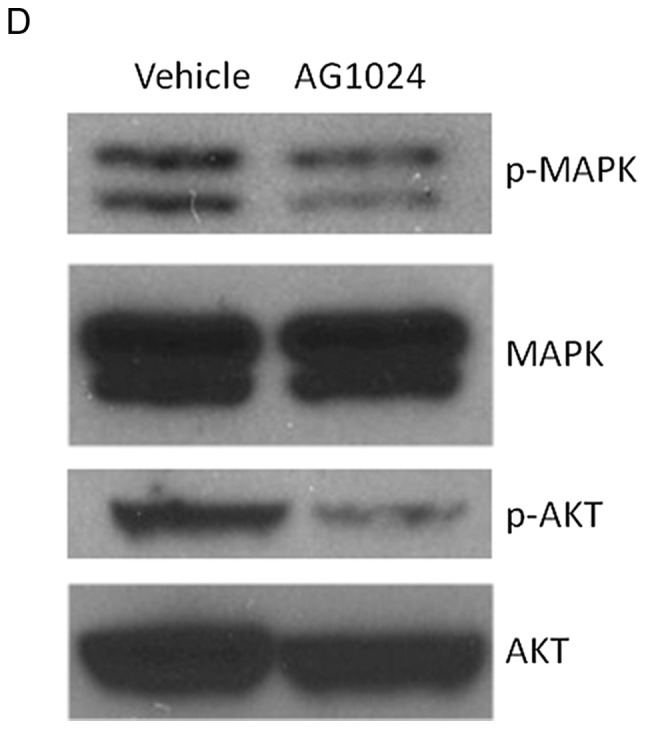
MCF-7:2A IGF-1Rβ. (A) Basal IGF-1Rβ mRNA was measured in MCF-7:5C cells and MCF-7:2A cells via RT-PCR. MCF-7:2A values are normalized to MCF-7:5C. 36B4 was used as an internal control. Means represent samples in triplicate. (B) Basal IGF-1Rβ protein levels were measured in MCF-7:5C and MCF-7:2A cells by western blot analysis. β-actin was used as a loading control. (C) MCF-7:2A cells were treated with vehicle, 1 nM E_2_, 10 *μ*M AG1024, or 1 nM E_2_ + 10 *μ*M AG1024. DNA was harvested and quantified after seven days. Means represent samples in triplicate. (D) MCF-7:2A cells were treated for 72 h with vehicle or 10 *μ*M AG1024. Growth pathway protein levels were visualized via western blot analysis. Total MAPK and total AKT were used as loading controls. ^*^P<0.05, ^***^P<0.001.

Insulin-like growth factor receptor β (IGF-1Rβ) upregulation is another mechanism through which MCF-7:2A cells could receive anti-apoptotic advantage over MCF-7:5C cells. MCF-7:2A cells exhibit 2.71-fold greater basal IGF-1Rβ mRNA than MCF-7:5C cells (Fig. 7A). This is consistent at the protein level as shown by western blot analysis, where MCF-7:2A cells exhibit more IGF-1Rβ protein expression than MCF-7:5C cells (Fig. 7B). When treated with an IGF-1Rβ inhibitor (10 *μ*M AG1024) for 7 days, MCF-7:2A cells show significantly decreased DNA content when compared to vehicle and 1 nM E_2_ treatments (Fig. 7C). Combination treatment of 1 nM E_2_ + 10 *μ*M AG1024 decreased DNA content significantly more than either treatment alone (Fig. 7C), suggesting an integral role of IGF-1Rβ in MCF-7:2A cells evading E_2_-induced apoptosis. To interrogate this further, growth pathway proteins were measured in response to 10 *μ*M AG1024 treatment. MAPK and AKT pathways are both blocked by the IGF-1Rβ inhibitor after 72 h as shown by decreased p-MAPK and p-AKT levels when compared to vehicle-treated MCF-7:2A cells (Fig. 7D).

## Discussion

This study investigated the mechanisms through which MCF-7:2A cells evade E_2_-induced apoptosis *in vitro* as a means to understand resistant breast cancer cells after long-term anti-hormone therapy in the clinic. After failure on an aromatase inhibitor, approximately 30% of breast cancer patients will respond to treatment with E_2_ (10); their nascent or remaining breast tumors will become cytostatic or disappear with physiological levels of E_2_. Further, E_2_ replacement therapy (ERT) has been shown to reduce the risk of breast cancer in hysterectomized post-menopausal women (12), perhaps due to E_2_-deprived breast cancer cells undergoing E_2_-induced apoptosis before resulting in clinically apparent disease. This study sought to discriminate between E_2_-deprived breast tumors that will quickly respond to treatment with E_2_ versus those that will respond more slowly and less dramatically. We modeled these different scenarios with MCF-7:5C and MCF-7:2A cell lines, respectively.

We have found that the UPR, associated with endoplasmic reticulum stress (ERS), is a fundamental element in E_2_-induced MCF-7:5C cell apoptosis (8). In this setting, E_2_ triggers UPR and rapidly causes apoptosis within one week of treatment. Two main sensors of the UPR, IRE1α and PERK are activated in both cell lines similarly. PERK activation is confirmed by elevated p-eIF2α, since eIF2α is phosphorylated by activated PERK. In MCF-7:2A cells, the same sensors are activated as in MCF-7:5C cells (Fig. 3), but significant cell death is not apparent at the same timepoint (Fig. 2A). Despite similar signaling patterns, the biological responses between the two cell lines differ. Our data suggested that another mechanism was preventing cell death after E_2_-induced UPR in MCF-7:2A cells.

Oxidative stress is a critical pathway for MCF-7:2A cells to undergo E_2_-induced apoptosis. MCF-7:2A cells inherently exhibit stronger survival and antioxidant mechanisms than MCF-7:5C cells (Figs. 4–6). This relationship is consistent with previously published data showing that MCF-7 cells with higher levels of glutathione peroxidase-1 (GSHPx-1) can survive better under oxidative stress conditions, such as hydrogen peroxide treatment (13), and that MCF-7 cells can increase antioxidant enzymes (i.e. manganese superoxide dismutase, MnSOD) to prevent TNF-mediated apoptosis (14). Activation of E_2_-induced apoptosis in MCF-7:2A cells also seems to require TNF family member upregulation (Fig. 4A and B). Oxidative stress occurs concurrently with upregulation of apoptosis-related genes in the TNF family. Whether increased TNFα causes oxidative stress or oxidative stress causes increased TNFα is not yet documented in this setting.

Additionally, B cell lymphoma 2 (BCL2) plays a role in preventing cell death caused by oxidative stress (15). In fact, MCF-7:2A cells exhibit 3.76-fold and 3.02-fold higher basal BCL2 and B cell lymphoma extra large (BCL-xL, BCL2L1) mRNA levels than MCF-7:5C cells, respectively (Table I), providing support for the idea of a stronger survival signal. Other data from our lab shows that MCF-7:2A cells exhibit 6.19-fold higher glutathione peroxidase 2 gene (GPX2) over MCF-7:5C cells (Table II), illustrating more evidence in favor of increased protection from E_2_-induced oxidative stress and apoptosis in this context.

Increased IGFR promotes anti-hormone resistance in breast cancer, likely through growth factor receptor crosstalk and aberrant ER, MAPK, and AKT signal transduction pathway activation (16–18). Our data correlate with these findings in that higher IGF-1Rβ mRNA and protein expression confer a growth advantage and apoptotic resistance in MCF-7:2A cells despite treatment with E_2_ (Fig. 7). This suggests an IGF-1Rβ signaling pathway that can circumvent normal ER signaling in long-term estrogen-deprived breast cancer cells. Studies using hepato-cellular carcinoma cells (HCC) have demonstrated that IGF-1R overexpression can potentially cause increased glutathione transferase (GST) and protection from oxidative stress (19). Although this mechanism is shown in liver cancer cells, it may apply to our models of breast cancer as well. Perhaps the higher level of IGF-1Rβ in MCF-7:2A cells generates the increased glutathione levels necessary to escape cell death in the presence of E_2_.

The evidence thus far shows that TNF family member gene expression, protection against oxidative stress, and growth factor signaling are major mechanisms underlying the different biological responses to E_2_ seen in MCF-7:2A cells versus MCF-7:5C cells. Despite similar UPR signaling patterns, MCF-7:2A cells resist ERS-induced death longer and stronger than MCF-7:5C cells. Additional studies may provide further insight into the connection between IGF-1Rβ and glutathione in MCF-7:2A cells, and how this relationship functions in the presence and absence of a stressor such as E_2_. In order to effectively treat breast cancer patients who have undergone exhaustive anti-hormone treatment, and to explain why ERT can prevent breast cancer in some post-menopausal women, the examination of breast cancer cell models of E_2_ deprivation is proving invaluable. By understanding mechanisms that prevent apoptosis in these breast cancer cells, we can translate key findings into clinical practice.

## Figures and Tables

**Table I.** Basal apoptosis gene expression in MCF-7:2A cells versus MCF-7:5C.

Gene symbol	Fold change
AIFM2	5.7601
AKT1	2.5203
ANXA1	57.2949
ANXA4	2.7965
APAF1	2.839
ATF5	2.5303
BAG1	2.7188
**BCL2**	**3.7598**
**BCL2L1**	3.0192
BDNF	5.8519
BIK	6.2803
BIRC7	33.6437
CARD9	2.7968
CASP7	2.5278
CD27	2.7439
CD5	3.884
CD70	8.1739
CRYAB	2.967
CUL3	3.2377
DAPK1	2.6145
DAPK2	6.023
EDAR	5.7874
ERCC3	2.7634
ERN2	5.1671
GRM4	6.4268
HTT	4.3186
HIP1	5.7736
HSPA1B	2.5548
HSPB1	7.5902
IGF1R	3.4421
IL1A	31.2667
INHA	2.5996
LGALS1	430.9062
MAL	3.0587
MALT1	3.2679
NLRC4	2.84
NOL3	2.9365
PLAGL1	3.3963
PLAGL2	3.0314
PPP1R13B	2.7465
PPP2R1B	4.5273
PRKCA	2.503
PRODH	3.8158
PTH	5.7472
PYCARD	3.1633
RARG	2.968
SEMA4D	2.9335
SFN	3.2245
SIPA1	3.777
SOCS2	4.3464
STK17B	3.8901
TBX5	3.3289
TNFRSF10D	2.5864
TNFRSF18	4.0067
TNFRSF19	76.9083
TNFRSF6B	2.7982
TNFRSF8	3.103
TNFSF14	4.5599
TP63	15.4118
TRAF2	2.5655
UNC13B	3.0047
VHL	3.1063
ZAK	2.8369

RT-PCR gene arrays of apoptosis-related genes were performed using MCF-7:5C and MCF-7:2A cells. Fold change represents gene expression of basal MCF-7:2A levels over basal MCF-7:5C levels. Only genes overexpressed in MCF-7:2A cells are shown. Particularly noteworthy in this study are BCL2 and BCL2L1.

**Table II.** Top 10 overexpressed and underexpressed oxidative stress-related genes in MCF-7:2A versus MCF-7:5C.

Gene name	Gene symbol	Category	Fold change
**Glutathione peroxidase 2**	***GPX2***	**Glutathione peroxidases, oxidative stress responsive genes**	**6.19**
Keratin 1	*KRT1*	Oxidative stress responsive genes	2.71
Heme oxygenase 1	*HMOX1*	Oxidative stress responsive genes	2.66
Thioredoxin reductase 1	*TXNRD1*	Oxidative stress responsive genes, other antioxidants	2.24
Peroxiredoxin 1	*PRDX1*	Peroxiredoxins (TPx)	2.22
24-Dehydrocholesterol reductase	*DHCR24*	Oxidative stress responsive genes	2.21
Aldehyde oxidase	*AOX1*	Other genes involved in ROS metabolism	2.20
Forkhead box M1	*FOXM1*	Oxidative stress responsive genes	1.83
Thioredoxin	*TXN*	Oxidative stress responsive genes	1.71
Prostaglandin-endoperoxide synthase 1	*PTGS1*	Other peroxidases	1.71
Copper chaperone for superoxide dismutase	*CCS*	Other genes involved in superoxide metabolism	−1.65
Ring finger protein 7	*RNF7*	Oxidative stress responsive genes	−1.65
Neutrophil cytosolic factor 2	*NCF2*	Other genes involved in superoxide metabolism	−1.81
NADPH oxidase, EF-hand calcium binding domain 5	*NOX5*	Other genes involved in superoxide metabolism	−1.83
Scavenger receptor class A, member 3	*SCARA3*	Oxidative stress responsive genes	−1.98
Superoxide dismutase 3, extracellular	*SOD3*	Superoxide dismutases, other antioxidants	−2.55
Cytochrome b-245, beta polypeptide	*CYBB*	Other peroxidases	−3.19
Selenoprotein P, plasma, 1	*SEPP1*	Oxidative stress responsive genes	−4.94
Apolipoprotein E	*APOE*	Oxidative stress responsive genes, other antioxidants	−8.55
Chemokine (C-C motif) ligand 5	*CCL5*	Oxidative stress responsive genes	−50.23

Global gene expression analyses were performed, and oxidative stress-related genes were ranked by fold change of MCF-7:2A expression over MCF-7:5C expression. Notably, GPX2 shows the highest fold change.
